# Supplementary material for: The causal relationship between the human gut microbiota and pyogenic arthritis: a Mendelian randomization study
Source: Front Cell Infect Microbiol. 2024 Nov 26;14:1452480. doi: 10.3389/fcimb.2024.1452480 (PMC11629706; doi:10.3389/fcimb.2024.1452480)
Supplement: Supplementary file 1 [file DataSheet1.zip › mendelian test/ebi-a-GCST90027513.csv_leave_one_out.pdf]

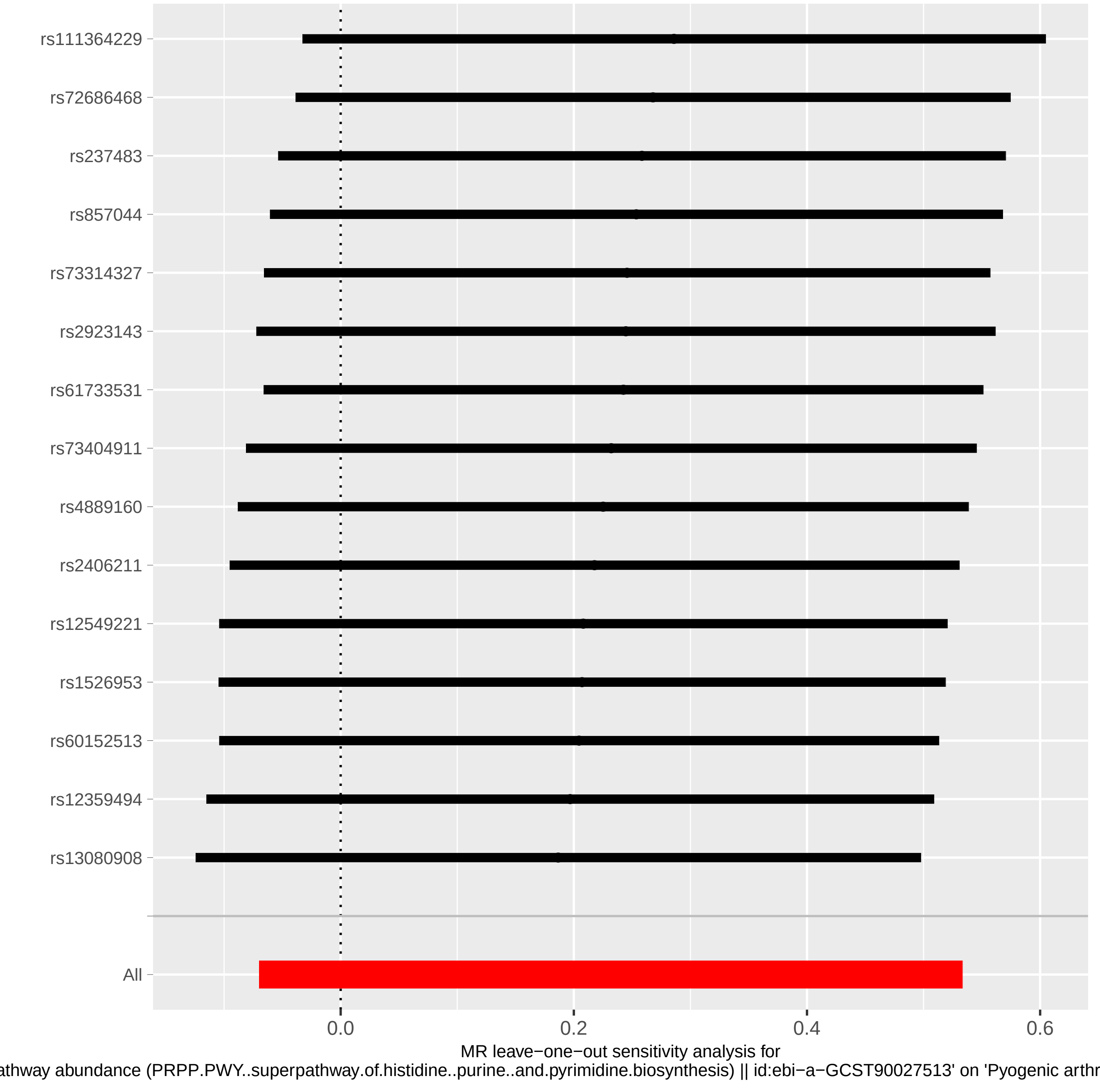
rs111364229
rs72686468
rs237483
rs857044
rs73314327
rs2923143
rs61733531
rs73404911
rs4889160
rs2406211
rs12549221
rs1526953
rs60152513
rs12359494
rs13080908
All
0.0
0.2
0.4
0.6
MR leave−one−out sensitivity analysis for
athway abundance (PRPP.PWY..superpathway.of.histidine..purine..and.pyrimidine.biosynthesis) || id:ebi−a−GCST90027513' on 'Pyogenic arthr
